# Supplementary material for: Quantification of free-living activity patterns using accelerometry in adults with mental illness
Source: Sci Rep. 2017 Mar 7;7:43174. doi: 10.1038/srep43174 (PMC5339808; doi:10.1038/srep43174)
Supplement: Supplementary Information [file srep43174-s1.pdf]

Title: Quantification of free-living activity patterns using accelerometry in adults with mental illness

Justin J. Chapman[1], **James A. Roberts**[1,2], **Vinh T. Nguyen**[1], **Michael Breakspear**[1,3]

[1] Systems Neuroscience Group, QIMR Berghofer Medical Research Institute, Brisbane, Queensland, Australia 4029.

[2] Centre for Integrative Brain Function, QIMR Berghofer Medical Research Institute, Brisbane, Queensland, Australia 4029.

[3] The Royal Brisbane and Women's Hospital, Brisbane, Queensland, Australia

Contents:

Table S1 – Diagnostic characteristics……………………………………………...page 2

Figure S2- CDFs using 60-second epoch accelerometer data………………. ...page 5

Table S2 - Hierarchical multiple regression to investigate the influence of participant characteristics, and activity pattern features, on the parameters of the successful group-wise model ……..page 6

Table S3 - Group-averaged parameters for waking data and sleep data ……..page 7

Table S1
Diagnostic characteristics of the analytic sample (n=99)

**Single diagnostic group (n=41)**	**n**
Bipolar (n=14)	
BP2	7
BP1	7
Psychosis (n=15)	
Schizophrenia	9
Psychotic disorder NOS	6
Mood (n=3)	
MDD	3
Anxiety (n=5)	
PTSD	2
GAD	1
OCD	1
PD	1
Substance dependence (n=4)	
Drug dependence	3
Alcohol & drug dependence	1
**Two diagnostic groups (n=33)**	**n**
Bipolar and anxiety (n=5)	
BP1 and PD	2
BP1, PD, and agoraphobia	1
BP2 and PD	1
BP2 and GAD	1
Bipolar and Substance (n=1)	
BP1 and drug dependence	1
Bipolar and Eating disorder (n=1)	
BP1 and bulimia nervosa	1
Psychosis and Mood (n=1)	
Psychotic disorder NOS and MDD	1
Psychosis and Anxiety (n=5)	
Schizophreniform disorder and agoraphobia	1

Schizophrenia, PD, and agoraphobia	1
Schizoaffective disorder and PD	1
Schizoaffective disorder, GAD, and OCD	1
Psychotic disorder NOS, and PTSD	1
Psychosis and Substance (n=4)	
Psychotic disorder NOS and drug dependence	3
Schizophrenia and drug dependence	1
Mood and Anxiety (n=8)	
MDD and GAD	3
MDD and PTSD	2
MDD and PTSD and GAD	1
MDD and agoraphobia	1
MDD, PD, PTSD and GAD	1
Mood and Substance (n=3)	
MDD and drug dependence	3
Mood and Eating disorder (n=1)	
MDD and bulimia nervosa	1
Anxiety and Substance (n=2)	
PD, PTSD, and drug dependence	1
PTSD, agoraphobia, and alcohol dependence	1
Anxiety and ADHD (n=1)	
Obsessive disorder, and ADHD	1
Substance and ADHD (n=1)	
Drug dependence and ADHD	1
**Three or more diagnostic groups (n=9)**	**n**
Bipolar, Anxiety, and Substance (n=3)	
BP2, PTSD, agoraphobia, and alcohol dependence	1
BP2, PTSD, and alcohol dependence	1
BP1, PTSD, OCD, agoraphobia, and alcohol dependence	1
Psychoses, Mood, Anxiety and Substance (n=1)	
Schizoaffective disorder, MDD, PD, agoraphobia, OCD, PTSD, and alcohol dependence	1
Psychoses, Mood and Substance (n=1)	
Psychoses NOS, MDD, alcohol and drug dependence	1

Psychosis, Anxiety, Substance, and ADHD (n=1)	
Psychotic disorder NOS, PD, GAD, alcohol & drug dependence, and ADHD	1
Psychoses, Substance and ADHD (n=1)	
Schizoaffective disorder, alcohol & drug dependence, and ADHD	1
Mood, Anxiety and Substance (n=2)	
MDD, PTSD and alcohol dependence	1
MDD,OCD, PTSD and alcohol dependence	1
None [a]	16

[a] Self-reported diagnoses of participants who did not screen positive for a current psychiatric diagnosis on the MINI-Plus were: psychoses (n=6), depression (n=4), depression and anxiety (n=2), depression and bipolar disorder (n=1), anxiety (n=1), bipolar disorder (n=1), and psychosis secondary to an acquired brain injury (n=1).

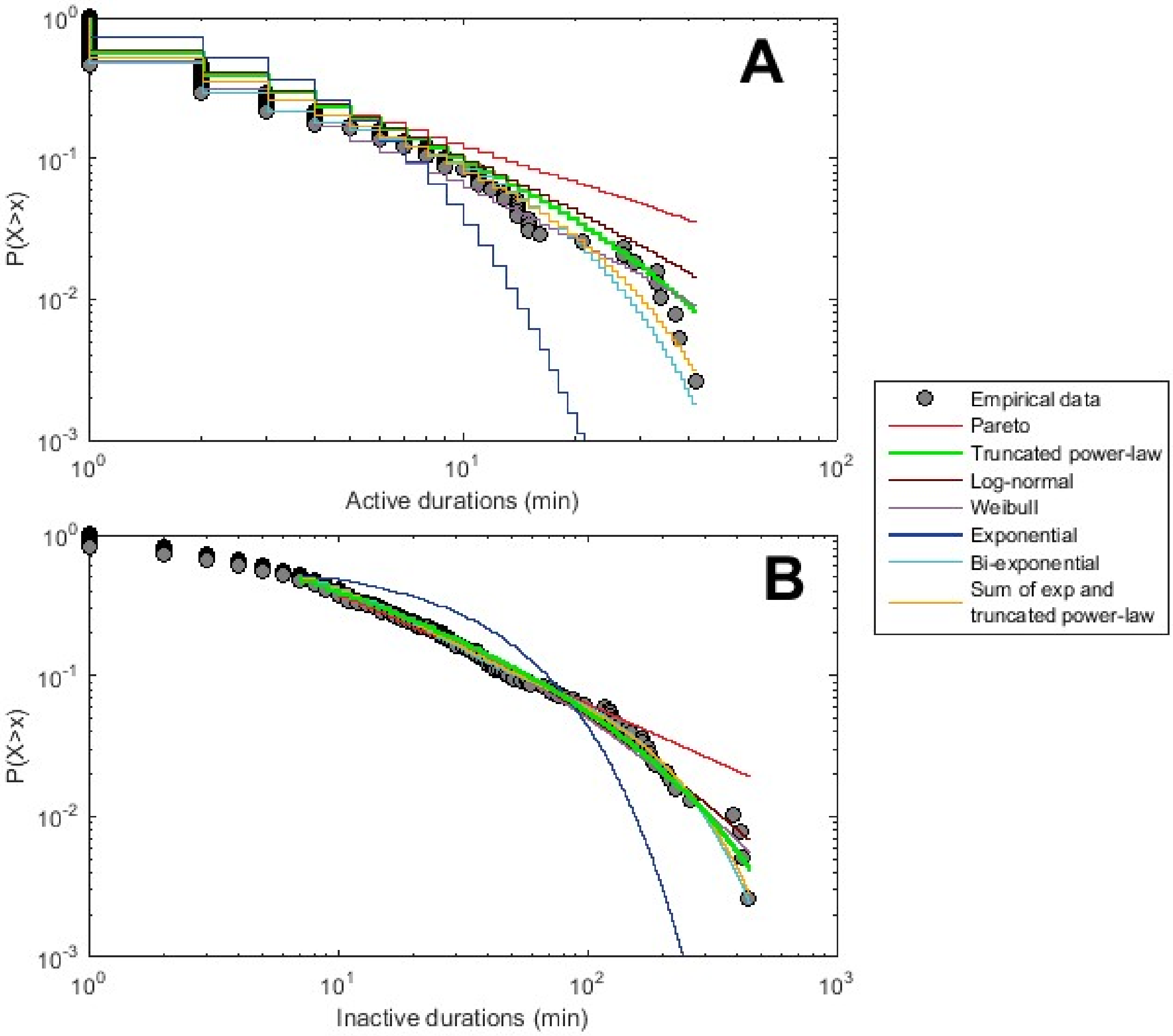

Figure S1 – Cumulative distribution functions (CDFs) and model fits for (**A**) active durations, and (**B**) inactive durations, using 60-second epoch accelerometer data. Truncated power-law was the most successful model for active (exceedance probability φ=1.0; parameters: α=2.09, λ=0.009) and inactive (exceedance probability φ=1.0; parameters: α=1.85, λ=0.003) CDFs.

Table S2

Hierarchical multiple regression to investigate the influence of participant characteristics, and activity pattern features, on the parameters of the successful group-wise model

Data	Independent variable	Significant predictors	Predictor statistics			Model			
			B (95%CI)	t	*p*	R2	F	*p*	Adjusted R2
Active periods	α	1. SB_PC	-0.019 (-0.0323, -0.007)	-3.064	0.003	0.097	F(2, 94) = 5.035	0.008	0.078
		2. MVPA_PC	-0.035 (-0.065, -0.005)	-2.287	0.024				
	λ (nat log transformed)	1. MVPA_PC	-0.129 (-.206, -0.052)	-3.316	0.001	0.105	F(1, 94) = 10.993	0.001	0.095
	γ	1. MVPA_PC	0.022 (0.011, 0.034)	3.930	<0.001	0.139	F(1, 96) = 15.442	<0.001	0.13
	δ	1. None	-	-	-	-	-	-	-
Dwell periods	α (nat log transformed)	1. SB_PC	-0.008 (-0.011, -0.005)	5.2	<0.001	0.271	F(3,90)=11.141	<0.001	0.247
		2. MVPA_PC	-0.012 (-0.019, -0.004)	3.199	0.002				
		3. Smoking status	0.031 (0.001, 0.06)	2.04	0.044				
	λ	1. Age	3.35e-6 (0, 0)	-3.062	.003	0.127	F(2,94)=6.867	0.002	0.109
		2. SB_PC	2.58e-6 (0, 0)	2.285	.025				

Note: SB_PC: average daily proportion of time spent in sedentary behavior; MVPA_PC: average daily proportion of time spent in moderate-to-vigorous physical activity; Smoking status: categorized as i) 'never or quit more than 6 months ago', ii) Occasionally (less than daily), iii) daily smoker.

The above results represent regression statistics for the variables that contributed significantly to the explanatory model (p<0.05)

Table S3

Bayesian model selection and group-averaged parameters for waking data (n=99) and sleep data (n=89) [a]

**Probability distributions**	**Active distributions**				**Inactive distributions**			
	**Waking data**		**Sleep data**		**Waking data**		**Sleep data**	
	Exceedance probability (φ)	Group-averaged parameters	Exceedance probability (φ)	Group-averaged parameters	Exceedance probability (φ)	Group-averaged parameters	Exceedance probability (φ)	Group-averaged parameters
Power-law	0.039	$\alpha=2.888$	0	$\alpha=3.053$	0.012	$\alpha=2.613$	$1\text{x}10^{-6}$	$\alpha=2.793$
Exponential	0	NA	**1.0**	$\boldsymbol{\lambda=0.521}$	0	NA	0	$\lambda=0.001$
Log-normal	0	$\mu=-1.822$<br>$\sigma=1.811$	0	$\mu=-9.817$<br>$\sigma=1.971$	0	$\mu=0.807$<br>$\sigma=2.067$	0	NA
Weibull	0	$\beta=0.108$<br>$\lambda=4.7\text{x}10^{-10}$	0	$\beta=0.263$<br>$\lambda=0.076$	0	$\beta=0.184$<br>$\lambda=0.560$	0	NA
Truncated power-law	0	$\alpha=2.085$<br>$\lambda=0.009$	$7\text{x}10^{-6}$	$\alpha=2.310$<br>$\lambda=0.123$	**0.988**	$\boldsymbol{\alpha=1.65}$<br>$\boldsymbol{\lambda=5.6\text{x}10^{-4}}$	$2.2\text{x}10^{-5}$	$\alpha=1.289$<br>$\lambda=2.0\text{x}10^{-4}$
Biexponential	0	$\lambda=0.406$<br>$\gamma=0.022$<br>$\delta=0.939$	0	$\lambda=0.507$<br>$\gamma=0.268$<br>$\delta=0.871$	0	$\lambda=0.015$<br>$\gamma=0.002$<br>$\delta=0.508$	0	$\lambda=4.7\text{x}10^{-4}$<br>$\gamma=0.007$<br>$\delta=0.536$
Sum of exponential and truncated power-law	**0.961**	$\boldsymbol{\alpha=1.735}$<br>$\boldsymbol{\lambda=0.030}$<br>$\boldsymbol{\gamma=0.472}$<br>$\boldsymbol{\delta=0.434}$	0	NA	$3.3\text{x}10^{-5}$	$\alpha=1.516$<br>$\lambda=0.006$<br>$\gamma=0.002$<br>$\delta=0.139$	**1.0**	$\boldsymbol{\alpha=1.242}$<br>$\boldsymbol{\lambda=0.012}$<br>$\boldsymbol{\gamma=6.9\text{x}10^{-4}}$<br>$\boldsymbol{\delta=0.219}$

Note: Models with group-averaged parameters of "NA" were not potentially plausible models for any cases; plausible models were determined from the log-evidence ratios of at least 0.05.

[a] Ten participants had insufficient sleep data, so were not included in analysis of pooled waking and sleeping data.
